# Clinical Characteristics and Correlates of Illness Severity in a Clozapine-Treated Cohort: A Service Evaluation From Suffolk

**DOI:** 10.1192/bjo.2026.11617

**Published:** 2026-06-30

**Authors:** Nada Zahreddine, Lucy Mountford, Sarah Zahreddine, Ahmed Ibrahim, Angelos Zikos, Mohamed Elkoshiery, Maria Giner-Murillo

**Affiliations:** 1 Norfolk and Suffolk NHS Foundation Trust, Ipswich, United Kingdom; 2 University of Geneva, Geneva, Switzerland; Mohamed.elkoshiery@nsft.nhs.uk, ST, maria.giner-murillo@nsft.nhs.uk, CT NSFT, Ipswich, UK

## Abstract

**Aims:**

Clozapine is the gold-standard treatment for treatment-resistant schizophrenia. Its requirement for regular blood testing and monitoring has led to centralised clozapine clinics in the UK, yet real-world data remain limited.

**Methods:**

This cross-sectional service evaluation examined patients attending a clozapine clinic serving adult secondary care services across Suffolk. Demographic, clinical, and service-provision data were extracted from electronic health records. Illness severity was assessed using the Clinical Global Impression–Schizophrenia scale (CGI-SCH), and functioning using the Global Assessment of Functioning (GAF).

**Results:**

The cohort comprised 257 patients (mean age 46.9 years, SD=12.4), predominantly male (68.9%) and largely White British (78.0%); 35.2% were current smokers. Meanclozapine dose was 330.4 mg/day (SD=134.0), with a mean treatment duration of 12.4 years (SD=7.8). In the prior year, 81.7% attended the clinic, 48.2% had at least one clozapine plasma level recorded, 12.1% had an inpatient admission, 32.7% had an allocated care coordinator, and patients attended a mean of 2.7 psychiatrist appointments (SD=1.3).

In addition to clozapine, 26.5% were prescribed an additional antipsychotic (most commonly aripiprazole 14.8% and amisulpride 11.7%). The most frequently prescribed mood stabilisers were valproate (11.7%) and lithium (3.9%). Antidepressants were prescribed in 44.4%, and benzodiazepines in 17.9%.

The most prevalent primary diagnoses were schizophrenia (86.4%) and schizoaffective disorder (10.1%). Common psychiatric comorbidities included autism spectrum disorder/learning disability (ASD/LD; 14.8%), anxiety disorders (12.5%), obsessive–compulsive disorder (5.4%), and major depressive disorder (MDD; 4.3%).

Mean CGI-SCH scores indicated low overall symptom severity (CGI-Severity M=2.28, SD=1.17). Mean GAF score was 61.1 (SD=14.6), consistent with mild–moderate functional impairment. Symptomatic remission (CGI ≤ 3) was observed in 85.4%, and functional remission (CGI ≤ 3 and GAF ≥ 60) in 55.5%.

Male gender was associated with greater negative symptom severity (p=.045), while female gender was associated with greater depressive symptom severity (p=.045) and antidepressant prescribing (p=.004).

Multivariable linear regression predicting CGI-Severity was statistically significant (p < .001). After adjustment for demographic, clinical, and treatment-related variables, higher illness severity was independently associated with schizoaffective disorder, MDD, ASD/LD, higher clozapine dose, shorter clozapine treatment duration, recent inpatient admission, and recent changes to clozapine dosing (all p ≤ .030).

**Conclusion:**

Clozapine-treated patients showed high rates of symptomatic remission, yet illness severity was independently associated with diagnostic complexity and markers of clinical instability, highlighting targets for enhanced, proactive service input.

